# Association between alcohol consumption and peripheral artery disease: two *de novo* prospective cohorts and a systematic review with meta-analysis

**DOI:** 10.1093/eurjpc/zwae142

**Published:** 2024-04-16

**Authors:** Shuai Yuan, Jing Wu, Jie Chen, Yuhao Sun, Stephen Burgess, Xue Li, Agneta Åkesson, Susanna C Larsson

**Affiliations:** Institute of Environmental Medicine, Karolinska Institutet, Nobels väg 13, Stockholm 17177, Sweden; Institute of Environmental Medicine, Karolinska Institutet, Nobels väg 13, Stockholm 17177, Sweden; School of Public Health, Zhejiang University School of Medicine, Hangzhou, China; School of Public Health, Zhejiang University School of Medicine, Hangzhou, China; MRC Biostatistics Unit, University of Cambridge, Cambridge, UK; Department of Public Health and Primary Care, University of Cambridge, Cambridge, UK; Department of Big Data in Health Science School of Public Health, Center of Clinical Big Data and Analytics of The Second Affiliated Hospital, Zhejiang University School of Medicine, Hangzhou, China; Institute of Environmental Medicine, Karolinska Institutet, Nobels väg 13, Stockholm 17177, Sweden; Institute of Environmental Medicine, Karolinska Institutet, Nobels väg 13, Stockholm 17177, Sweden; Department of Surgical Sciences, Uppsala University, Uppsala, Sweden

**Keywords:** Alcohol, Cohort, Meta-analysis, Peripheral artery disease

## Abstract

**Aims:**

The association between alcohol consumption and risk of peripheral artery disease (PAD) is inconclusive. We conducted this study to examine the association between alcohol consumption and PAD risk in two *de novo* cohort studies and a meta-analysis of observational studies.

**Methods and results:**

A systematic review was conducted to identify studies on alcohol consumption in relation to PAD risk. We further used data from two cohorts of 70 116 Swedish and 405 406 British adults and performed a meta-analysis of results from previously published studies and current cohort studies. There was a U-shaped association between alcohol consumption and incident PAD risk in the Swedish and British cohorts. The meta-analysis of results of these two cohorts and previously published studies found that compared with non- or never-drinkers, the relative risk of PAD was 0.83 [95% confidence interval (CI) 0.77–0.89], 0.81 (95% CI 0.74–0.90), and 0.94 (95% CI 0.83–1.07) for light, moderate, and high-to-heavy alcohol drinkers, respectively. The nonlinear meta-analysis revealed a possibly U-shaped association between alcohol consumption and PAD risk (*P* nonlinearity <0.001). The risk of PAD was observed to be the lowest for 2 drinks/week and to be pronounced for ≥10 drinks/week. All these associations persisted in a sensitivity meta-analysis including cohort and other types of observational studies.

**Conclusion:**

Alcohol intake ≤2 drinks/week was associated with a reduced risk of PAD, and the risk of PAD became pronounced with intake ≥10 drinkers/week.

## Introduction

Peripheral artery disease (PAD) is a common atherosclerotic vascular disease that affects around 200 million individuals globally and associates with high rates of cardiovascular events and death.^1^ Among modifiable factors for PAD, smoking and diabetes have been identified as two strong causal risk factors,^2–4^ and consistent evidence from recent studies has also linked low adherence to a healthy diet and low levels of physical activity to an increased risk of this disease.^5,6^ Another possible modifiable lifestyle factor for PAD is alcohol consumption, but available data are inconclusive.^7^ Our cohort study based on 69 449 Swedish middle-aged adults found that lesser alcohol intake (≤2 drinks/day) was associated with a lower risk of PAD.^5^ In the PREDIMED study of 7122 individuals with high cardiovascular risk, moderate alcohol consumption (10–50 g/day in men or 5–25 g/day in women) was associated with a lower risk of PAD compared with low or high alcohol intake.^6^ However, a null finding of the association between any amount of alcohol consumption and PAD risk was reported in the Women’s Health Initiative study including 138 506 American postmenopausal women.^8^ Given that alcohol consumption is a common health-related behaviour and has inconsistent associations with PAD, an appraisal of the association between alcohol consumption and PAD risk is of great importance for the disease prevention.

## Methods

### Study design

**Figure 1:**
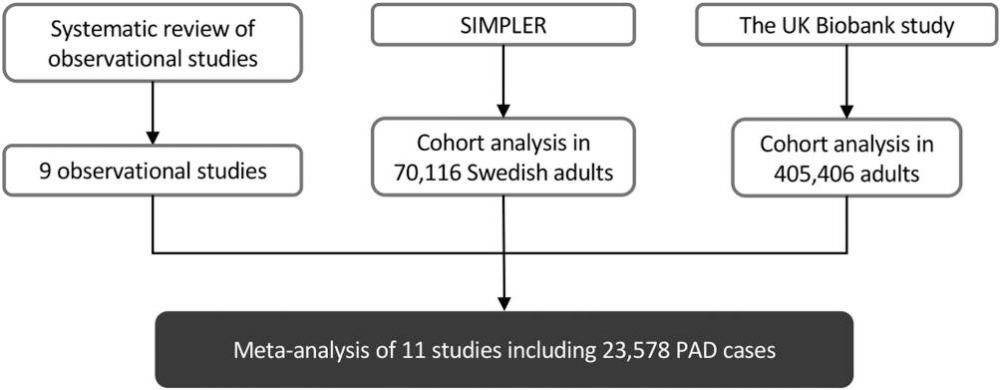
Study design overview. PAD, peripheral artery disease; SIMPLER, Swedish Infrastructure for Medical Population-Based Life-Course and Environmental Research.

The design of the present study is presented in *Figure 1*. We firstly conducted a systematic review of observational studies that examined the association between alcohol consumption and PAD risk. We then explored this association using data from the SIMPLER (Swedish Infrastructure for Medical Population-Based Life-Course and Environmental Research) and UK Biobank cohort studies. A meta-analysis of identified studies from systematic review, SIMPLER, and the UK Biobank was performed. The systematic review was conducted in accordance with the PRISMA^9^ and registered at https://www.crd.york.ac.uk/PROSPERO/ (PROSPERO: CRD42022367683).

### Systematic review

We searched the PubMed database from inception to 12 September 2023 using the search strategy presented in Supplementary Methods. No restrictions were imposed in the search process. We excluded studies by following the criteria: (i) inappropriate publication type (e.g. review articles); (ii) irrelevant to the research question; (iii) not in English; (iv) nonhuman study; (v) without effect estimate or enough data to calculate the effect estimate; and (vi) without adjustment for minimally required covariates, that is, age, sex, and smoking status (a strong risk factor for PAD and highly correlated with alcohol consumption). Due to a comparatively small number of studies identified, we set a relaxed criterion for minimally required covariates. For studies with sample overlap, the study with the larger sample size or longer follow-up was included. Two authors (S.Y. and J.W.) conducted the literature search and independently selected studies in a double-blind manner. Any disagreement was evaluated and resolved by a third senior author (S.C.L.). Likewise, S.Y. and J.W. used the Newcastle-Ottawa Scale^10^ to assess the quality of included studies, and any disagreements were solved by S.C.L. The Newcastle-Ottawa Scale examines three dimensions of a study, including selection, comparability, and outcome with a total score of 9. The higher the Newcastle-Ottawa score, the better the study quality. We extracted data on the name of the first author, study design, cohort name and location, participants, follow-up time (for cohort studies), number of cases, effect estimate [risk ratio, hazard ratio (HR), or odds ratio with 95% confidence intervals (CIs)] for each category of alcohol consumption, and covariates adjusted for the multivariable model. We unified the unit of alcohol consumption as drinks per week by assuming that one drink contains 12 g of alcohol.^11^ Nondrinker or never-drinker was considered the reference (comparison) group.

### SIMPLER

SIMPLER includes the Swedish Mammography Cohort and the Cohort of Swedish Men that were initiated in 1987 and 1997, respectively, and followed up. We used the 1997 data as the baseline given that two cohorts used same questionnaires with exception of certain sex-specific questions. Detailed description of the two cohorts can be found in our previous studies.^5,12^ After removing participants with baseline cancer and PAD, those without information on alcohol consumption, and past alcohol drinkers, 70 116 Swedish adults were included in the analysis. Information on alcohol consumption, including status (never, past, and current) and the amount consumed in grams per day, was obtained from the baseline self-administrated questionnaire. The baseline age was treated as a time scale. Additional covariates included sex, body mass index, education level, smoking, physical activity, diet quality, and baseline diagnosis of hypertension, hypercholesterolaemia, and diabetes with data obtained from the questionnaires (see Supplementary Methods). Incident PAD cases were defined using International Classification of Diseases codes (see Supplementary material online, *Table S1*) and identified by linkage of the cohorts to the Swedish Patient Register.^13^ Death information was obtained from the Swedish Death Registry. Individuals were followed up from 1 January 1998 until the date of diagnosis of PAD, date of death, or end of follow-up (i.e. 31 December 2019), whichever came first.

### UK Biobank

The UK Biobank is an ongoing cohort study that initially recruited >500 000 participants from 22 assessment centres across the UK between 2006 and 2010. We excluded individuals with baseline PAD diagnosis, with missing data on alcohol consumption, and past alcohol drinkers, leaving 405 406 individuals in the analysis. Alcohol consumption information was obtained from the baseline questionnaire. Peripheral artery disease cases were defined by a primary or secondary diagnosis with data on admissions and diagnoses and corresponding dates from hospital inpatient records using the International Classification of Diseases codes (see Supplementary material online, *Table S2*). The baseline age was treated as a time scale. Additional covariates included sex, ethnicity, body mass index, education level, Townsend deprivation index, smoking status, physical activity, diet quality, and baseline history of hypertension, diabetes, and hypercholesterolaemia. Detailed information on covariate definition is shown in Supplementary Methods. Death information was obtained from the Death Registry. Individuals were followed up from baseline (2006–2010) until the date of diagnosis of PAD, date of death, date of loss to follow-up, or the last date of hospital admission, whichever came first.

### Statistical analysis

In cohort analyses in SIMPLER and the UK Biobank, the Cox proportional hazard regression with age as the underlying time scale and covariate adjustment was used to obtain the associations of alcohol consumption with PAD risk. The nonlinearity of the association was examined by a restricted cubic spline model setting three knots at the 25th, 50th, and 75th percentiles of alcohol consumption. We used three models with different adjustments: Model 1 adjusted for sex and ethnicity (in the UK Biobank); Model 2 adjusted for sex, ethnicity (in the UK Biobank), body mass index, education level, smoking status, physical activity, and diet quality; and Model 3 (the main analysis) adjusted for sex, ethnicity (in the UK Biobank), body mass index, education level, smoking status, physical activity, diet quality, and baseline history of hypertension, diabetes, and hypercholesterolaemia. We conducted stratification analyses based on baseline age (<60 and ≥60 years), sex (men and women), and smoking status (never and ever smokers) in the *de novo* cohort analyses.

In meta-analysis, risk estimates (risk ratio, HR, and odds ratio) were interpreted as relative risks (RRs).^14^ The random-effects model was applied to combine the effect estimates according to three categories of alcohol intake: light (0.1–7 drinks/week), moderate (7.1–14 drinks/week), and high to heavy (≥14.1 drinks/week) that were determined by the average consumption in each category. Each study’s log-transformed risk estimate was weighted by the inverse of its variance and the between-study variance component *τ*^2^.^15^ The *I*^2^ statistic was used to quantify heterogeneity among studies,^16^ and 50% < *I*^2^ < 75% and *I*^2^ ≥ 75% were deemed moderate and high heterogeneity, respectively. The publication bias was assessed by funnel plot as well as Egger’s and Begg’s tests since the funnel plot may not detect publication bias when the number of studies is small. Finally, we conducted a nonlinear dose–response analysis by restricted cubic splines with three knots at 10, 50, and 90% percentiles of the distribution, which was combined using multivariate meta-analysis.^17^ The primary analysis was conducted by including only cohort studies to minimize reverse causality, and the secondary analysis was further performed by including cohort plus other types of observational studies to increase statistical power. All tests were two sided, and the analyses were performed in Stata/SE (version 15.0; StataCorp, TX, USA) and R software (version 4.0.2). A *P* value below 0.05 was deemed statistically significant.

## Results

### SIMPLER

The baseline characteristics of 70 116 participants by alcohol consumption are presented in Supplementary material online, *Table S3*. A total of 2554 PAD cases were diagnosed during a median follow-up period of 21.9 years. In Cox regression analysis, compared to never alcohol drinkers, the HR of PAD was 0.83 (95% CI 0.74–0.93), 0.82 (95% CI 0.71–0.94), 0.81 (95% CI 0.67–1.01), and 1.22 (95% CI 0.69–2.15) for light, moderate, high, and heavy alcohol drinkers, respectively (see Supplementary material online, *Table S4*). A U-shaped association was observed when treating alcohol consumption as a continuous variable in drinks per week in the restricted cubic spline Cox analysis (see Supplementary material online, *Figure S1*).

### UK Biobank

The baseline characteristics of 405 406 participants by alcohol consumption are presented in Supplementary material online, *Table S5*. A total of 4532 PAD cases were diagnosed during a median follow-up period of 11.8 years. Compared to never alcohol drinkers, the HR of PAD was 0.71 (95% CI 0.64–0.79), 0.70 (95% CI 0.62–0.79), 0.78 (95% CI 0.69–0.88), and 1.17 (95% CI 1.02–1.34) for light, moderate, high, and heavy alcohol drinkers, respectively (see Supplementary material online, *Table S6*). A U-shaped association between alcohol consumption in drinks per week and PAD risk was observed in the restricted cubic spline Cox analysis (see Supplementary material online, *Figure S2*).

### Stratification analysis in SIMPLER and UK Biobank

The association between alcohol consumption and PAD risk was generally consistent between baseline age groups in both cohorts (see Supplementary material online, *Tables S7* and *S8*). Likewise, the association was similar between subgroups by sex and smoking status in the UK Biobank (see Supplementary material online, *Table S8*). However, the inverse association between light-to-moderate alcohol intake and PAD risk was observed only among women and ever smokers in SIMPLER (see Supplementary material online, *Table S7*). Compared with never alcohol consumption, heavy alcohol consumption was associated with >100% higher risk of PAD in men in SIMPLER (HR = 2.03; 95% CI 1.21–3.42; Supplementary material online, *Table S7*).

### Systematic review and meta-analysis

A total of 9 out of 1112 studies identified from the systematic review met the inclusion criteria (see Supplementary material online, *Figure S3*). Information on the 9 included studies (4 cohort, 2 case–control, and 3 cross-sectional studies) is presented in *Table 1*.^8,18–25^ Quality assessments using the Newcastle-Ottawa Scale of these studies are presented in Supplementary material online, *Table S9*. The Newcastle-Ottawa score ranged from 3 to 8 out of 9. Only two studies were defined as high quality with the score of 8.

**Table 1:** Information on the nine included studies from the systematic review

Study	Design	Setting	Cases	Total	Follow-up	Sex	Alcohol consumption	Effect estimate
Camargo CA Jr et al., 1997	Cohort	USA	433	21 759	11 years	Both	Nondrinker	1.00 (reference)
							1–6 drinks/week	0.82 (0.64–1.05)
							≥7 drinks/week	0.74 (0.57–0.97)
Ogilvie RP et al., 2017	Cohort	USA	1569	14 082	19.9 years	Both	Nondrinker	1.00 (reference)
							1–6 drinks/week	0.78 (0.68, 0.89)
							≥7 drinks/week	0.89 (0.76, 1.04)
Bell S et al., 2017	Cohort	UK	11 519	1 937 360	6.0 years	Both	Nondrinker	1.00 (reference)
							Occasional	0.84 (0.83, 0.85)
							Moderate	0.82 (0.76, 0.88)
							Heavy	0.91 (0.90, 0.92)
Chen GC et al., 2021	Cohort	USA	1036	138 506	18.6 years	Women	Nondrinker	1.00 (reference)
							0–4.9 g/day	1.05 (0.82–1.33)
							5–14.9 g/day	1.06 (0.81–1.41)
							15–24.9 g/day	1.16 (0.83–1.62)
							≥25 g/day	1.17 (0.83–1.65)
Vliegenthart R *et al.*, 2002	Cross-sectional	The Netherlands	558	3975	—	Women	Nondrinker	1.00 (reference)
							≤10 g/day	0.70 (0.53, 0.91)
							10.1–19.9 g/day	0.66 (0.43, 1.00)
							>20 g/day	0.64 (0.41, 1.01)
						Men	Nondrinker	1.00 (reference)
							≤10 g/day	0.97 (0.58, 1.63)
							10.1–19.9 g/day	1.02 (0.57, 1.80)
							>20 g/day	0.97 (0.57, 1.65)
Xie X *et al.*, 2010	Cross-sectional	China	926	10 154	—	Men	Never-drinker	1.00 (reference)
							0.1–20 g/day	1.12 (0.55, 1.77)
							20.1–40 g/day	0.48 (0.24, 1.53)
							40.1–59.9 g/day	0.20 (0.11, 0.75)
							>60 g/day	2.88 (1.22, 4.02)
						Women	Never-drinker	1.00 (reference)
							0.1–20 g/day	0.60 (0.34, 1.83)
							20.1–40 g/day	0.89 (0.41, 1.52)
							40.1–59.9 g/day	0.77 (0.30, 1.82)
							>60 g/day	1.30 (0.90, 2.64)
Desormais I *et al.*, 2015	Cross-sectional	Central Africa	277	1871	—	Both	Nondrinker	1.00 (reference)
							Occasional	0.63 (0.44, 0.99)
							Regular	1.28 (0.67, 2.44)
Ciccarone E *et al.*, 2003	Case–control	Italy	144	432	—	Both	Never-drinker	1.00 (reference)
							≤12 g/day	0.92 (0.51, 1.67)
							13–35.9 g/day	1.00 (0.33, 2.76)
							≥36 g/day	0.95 (0.33, 2.76)
Yang S *et al.*, 2017	Case–control	China	30	476	—	Both	Nondrinker	1.00 (reference)
							≤64 g/day	2.07 (0.78–5.54)
							>64 g/day	6.35 (1.78–22.65)

One drink equals 12 g of ethanol. For categorical dosage, we assume that light corresponds to 0.1–7 drinks/week, moderate 7.1–14 drinks/week, and high-to-heavy >14.1 drinks/week. In Bell S *et al.*, 2017, occasional category was deemed light category. In Desormais I *et al.*, 2015, occasional and regular category was deemed light and moderate category, respectively.

**Figure 2:**
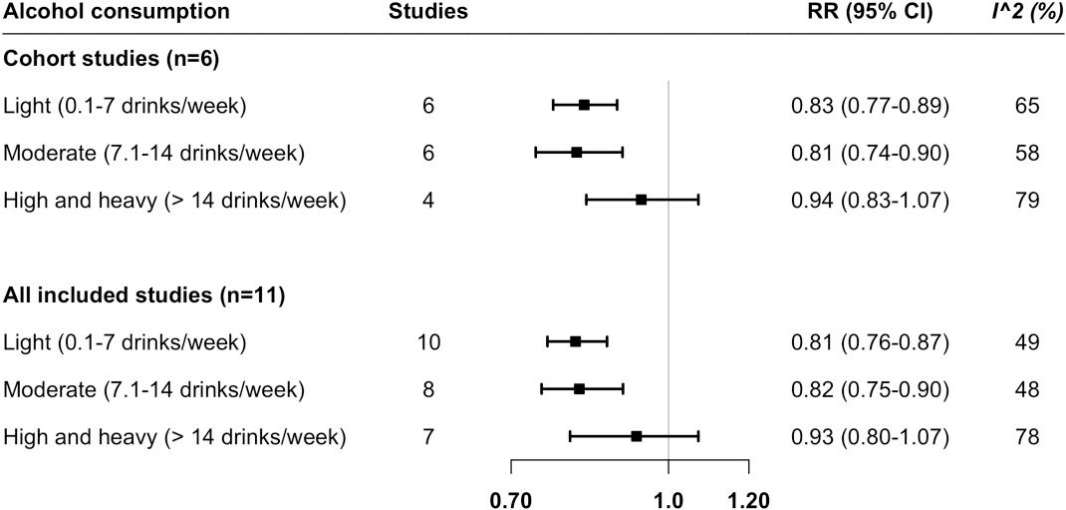
Association between alcohol consumption and peripheral artery disease risk in the meta-analysis of 11 observational studies. The reference group was nondrinkers or never-drinkers. CI, confidence interval; RR, risk ratio.

In the main meta-analysis of results from all cohort studies along with the SIMPLER and UK Biobank, including up to 21 643 PAD cases, light and moderate alcohol consumption but not high-to-heavy alcohol consumption was associated with a decreased risk of PAD (*Figure 2*). Compared with non- or never-drinkers, the RR of PAD was 0.83 (95% CI 0.77–0.89) for light alcohol drinkers, 0.81 (95% CI 0.74–0.90) for moderate alcohol drinkers, and 0.94 (95% CI 0.83–1.07) for high-to-heavy alcohol drinkers. The associations remained consistent in the sensitivity meta-analysis of all 11 studies (*Figure 2*). Moderate to high heterogeneity was observed in these meta-analyses (*Figure 2*). No sign of asymmetry was detected by funnel plots, Egger’s tests, or Begg’s tests (see Supplementary material online, *Figure S4*).

**Figure 3:**
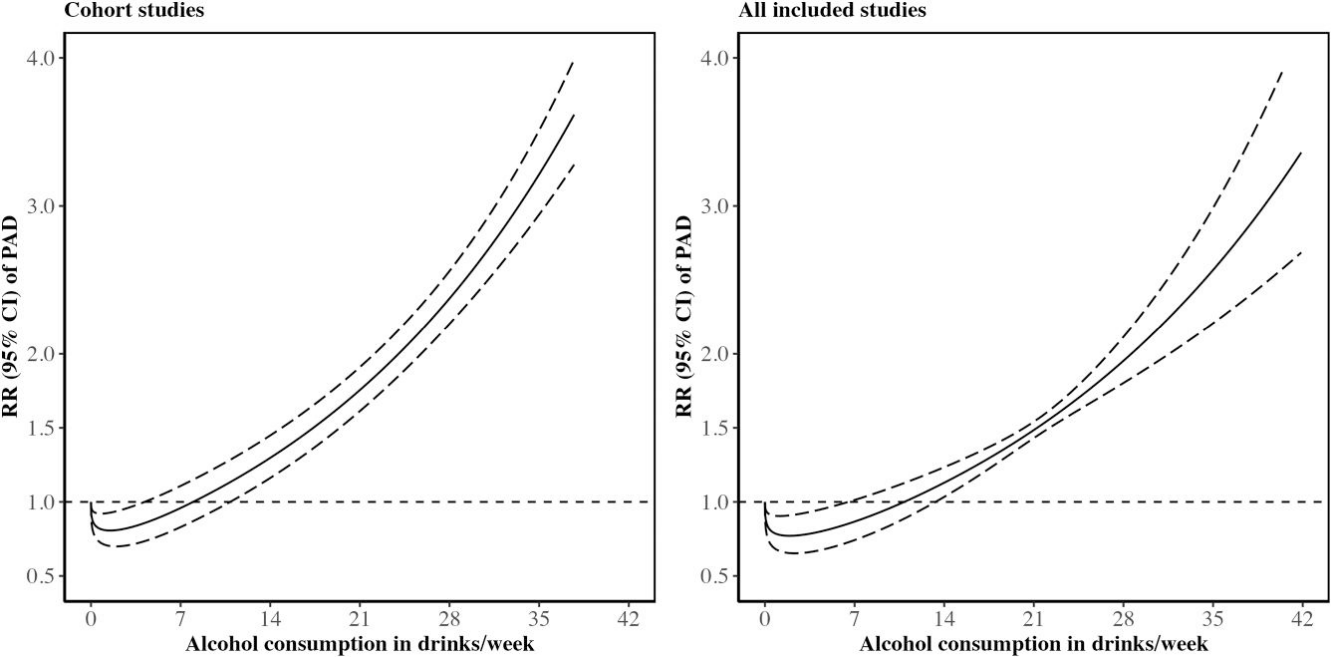
The nonlinear dose–response association between alcohol consumption and the risk of peripheral artery disease in the meta-analysis. CI, confidence interval; PAD, peripheral artery disease; RR, risk ratio.

The nonlinear dose–response analysis detected a possibly U-shaped association between alcohol consumption and the risk of PAD (*P* nonlinearity < 0.001; *Figure 3*). The risk of PAD was observed to be the lowest for individual with alcohol consumption of 2 drinks/week (the alcohol consumption level corresponding to the lowest RR) and started to be pronounced for individual with alcohol consumption ≥10 drinks/week (the alcohol consumption level corresponding to the RR with lower limit of 95% CI ≥ 1). The association remained consistent in the sensitivity meta-analysis of including all studies (*Figure 3B*).

## Discussion

The present study explored the association between alcohol consumption and risk of PAD using two *de novo* cohort analyses plus a systematic review with a meta-analysis. We found that light and moderate but not high-to-heavy alcohol consumption were inversely associated with the risk of PAD. The nonlinear (U-shaped) association between alcohol consumption and PAD risk was detected with the lowest risk for 2 drinks/week and the increased risk for ≥10 drinks/week.

### Comparison with previous studies

Alcohol consumption has been associated with cardiovascular disease risk; however, the shape of the association has not been consistently determined. In epidemiological studies, light-to-moderate alcohol consumption has been associated with a lower risk of coronary heart disease (CHD),^26^ heart failure,^27^ ischaemic stroke,^28^ and cardiovascular mortality.^29^ Recent studies also confined the association of light-to-moderate alcohol consumption with lower risk of certain cardiovascular outcomes, like majorly for CHD and myocardial infarction,^30^ and possibly to certain age groups, like individuals aged over 40 years and older.^31^ To our knowledge, no studies have systematically examined the association of alcohol consumption with PAD risk. Our first meta-analysis on this topic found an inverse association between light-to-moderate alcohol consumption and PAD risk, which is in line with evidence from large-scale updated studies^30,31^ since PAD shares the pathological basis (i.e. atherosclerosis) with CHD as well as the current study was based on the middle-aged and older populations. However, more and more genetic studies found a positive linear association between alcohol consumption and cardiovascular risk, which is against alcohol consumption at any levels for cardiovascular benefits.^32,33^

Binge or heavy alcohol consumption has been associated with an increased risk of cardiovascular events and death.^27,28,30^ Even though our categorical meta-analysis did not detect a clear positive association between high-to-heavy alcohol consumption and PAD risk, the nonlinear meta-analysis suggested that the protective effect of alcohol consumption on PAD was likely to disappear in people with >5 drinks/week and the risk became pronounced in those with ≥10 drinks/week.

### Potential mechanisms

The biological mechanisms in support of the protective role of light-to-moderate alcohol consumption in PAD are unclear but possibly and partly related to a lower risk of peripheral atherosclerosis and thrombosis due to the reduced levels of inflammation, oxidative stress, and thrombosis in light and moderate drinkers.^34^ Alcohol consumption was associated with microvascular complications in a U-shaped manner,^35^ which may also biologically support our findings given that microvascular dysfunction is another important pathological basis of PAD.^36^ Alcohol consumption has been found to influence skeletal muscle mass and capability,^37,38^ which impact the development of PAD.^36^ In addition, lower activity of a stress-related brain network among light-to-moderate alcohol consumers may also partly explain the inverse association with PAD.^39^

### Clinical and public health implications

Even though the study observed an inverse association between moderate alcohol consumption and PAD risk, it is not recommended to initiate alcohol consumption for abstainers to achieve this moderate beneficial impact on PAD after weighing against its potential adverse impacts on cancer,^39^ brain structure and activity,^40^ and substance dependency.^39^ For individuals with alcohol consumption ≥10 drinks per week, it is highly recommended to reduce alcohol intake to lower the corresponding risk of PAD as well as other health adversities.

### Strengths and limitations

A strength of this study is that we explored the association between alcohol consumption and PAD risk in two large-scale cohorts and further examined the association in a systematic review with a meta-analysis of a larger number of cases. Thus, we had more statistical power to estimate the association compared to individual studies. In addition, the associations were consistent between the analyses including only cohorts and cohorts plus other types of observational studies.

Several limitations need to be acknowledged when interpreting our results. First, the included studies were observationally designed. Thus, the observed association in the meta-analysis might be affected by confounding even though most studies controlled for important risk factors for PAD. In addition, confounding might be introduced since we used a comparatively relaxed ‘minimally required covariate’ selection strategy in the systematic review. However, quite consistent results were observed in the *de novo* prospective analyses using data from the UK Biobank and Swedish cohorts where the analyses were adjusted for more potential confounders. Second, we were not able to assess the validity of a self-administrated questionnaire for assessing alcohol consumption in all included studies. Nevertheless, 4 out of 11 included studies had corresponding validation studies for alcohol consumption.^20,41–43^ The correlation coefficients between alcohol intake assessed by the food frequency questionnaire and that from the several-day dietary interview or food records ranged between 0.81 in SIMPLER^43^ and 0.92 in Ciccarone *et al.*^20^ study. Even though no such validation study was conducted for the UK Biobank, a study found a high correlation (*r* = 0.99) for alcohol intake between repeated measurements.^44^ Thus, the validity of self-administrated questionnaire for assessing alcohol consumption should be satisfied. Third, although alcohol intake data obtained from the food questionnaires were valid, there might be measurement error in alcohol consumption caused by underreporting especially among heavy drinkers. However, the association persisted in the meta-analysis of cohort studies where misclassification of alcohol consumption would attenuate the true association in a conservative way assuming the misclassification of alcohol consumption was nondifferential due to the prospective design. Fourth, the study found potential sex difference in SIMPLER but not in UK Biobank. Thus, future studies are needed to differentiate the association by sex given that the cut-off and health effects of alcohol consumption may vary between women and men. Fifth, the study focused on overall alcohol consumption and did not examine the association across different types of alcoholic beverages. For example, intake of moderate levels of wine instead of liquor has been associated with a lower risk of cardiovascular disease since the enriched antioxidants of wine may decelerate atherogenesis.^45^ Sixth, most studies were based on middle-aged and older populations, which confined the generalizability of our findings to younger individuals. Last, most of the included studies had comparatively low quality.

In summary, light-to-moderate consumption of alcohol (≤2 drinks/week) was associated with a reduced risk of PAD in this meta-analysis of observational studies. The study further indicated a U-shaped association between alcohol consumption and PAD risk with the risk of PAD becoming pronounced for alcohol consumption >10 drinks/week. Taking all potential health consequences of alcohol intake into consideration, it is not recommended to initiate alcohol drinking for abstainers; instead, individuals who intake >10 drinks/week should lower their consumption to alleviate the risk of PAD and other health consequences.

## Supplementary Material

zwae142_Supplementary_Data

## Data Availability

De-identified SIMPLER data are available for researchers upon application (http://www.simpler4health.se/). Access to the UK Biobank data can be obtained upon application (https://www.ukbiobank.ac.uk/).
