# Supplementary material for: Association between alcohol consumption and peripheral artery disease: two de novo prospective cohorts and a systematic review with meta-analysis
Source: Eur J Prev Cardiol. 2024 Apr 16;32(2):149–55. doi: 10.1093/eurjpc/zwae142 (PMC7616826; doi:10.1093/eurjpc/zwae142)

Supporting information for

**Association between alcohol consumption and peripheral artery disease: two de novo prospective cohorts and a systematic review with meta-analysis**

*Shuai Yuan, Jing Wu, Jie Chen, Yuhao Sun, Fengzhe Xu, Agneta Åkesson, Stephen Burgess, Xue Li, Susanna C. Larsson*

Table of Contents

[Supplementary Methods 2](#_Toc161649473)

[Supplementary Table 1. Diagnostic information for peripheral artery disease in SIMPLER 4](#_Toc161649474)

[Supplementary Table 2. Diagnostic information for peripheral artery disease in UK Biobank 5](#_Toc161649475)

[Supplementary Table 3. Baseline characteristics of 70,116 participants by alcohol consumption status in SIMPLER 6](#_Toc161649476)

[Supplementary Table 4. Associations of alcohol consumption with incident PAD in SIMPLER 7](#_Toc161649477)

[Supplementary Table 5. Baseline characteristics of 405,406 participants by alcohol consumption status in the UK Biobank study 8](#_Toc161649478)

[Supplementary Table 6. Associations of alcohol consumption with incident PAD in the UK Biobank study 9](#_Toc161649479)

[Supplementary Table 7. Association between alcohol consumption and PAD risk by baseline age, sex, and smoking status in SIMPLER 10](#_Toc161649480)

[Supplementary Table 8. Association between alcohol consumption and PAD risk by baseline age, sex, and smoking status in the UK Biobank 11](#_Toc161649481)

[Supplementary Table 9. Quality assessment of included 9 studies. 12](#_Toc161649482)

[Supplementary Figure 2. The U-shaped association between alcohol consumption and PAD risk in the UK Biobank study 14](#_Toc161649483)

[Supplementary Figure 3. Flow chart of systematic review 15](#_Toc161649484)

# **Supplementary Methods**

**Search strategy (**#1 AND #2**)**

#1 "ethanol"[MeSH Terms] OR "alcohols"[MeSH Terms] OR "alcohol drinking"[MeSH Terms] OR "alcohol intake"[Title/Abstract] OR "alcohol drinking"[Title/Abstract] OR "alcohol"[Title/Abstract] OR "ethanol"[Title/Abstract] OR "alcohol consumption"[Title/Abstract]

#2 "peripheral arterial disease"[MeSH Terms] OR "peripheral vascular diseases"[MeSH Terms] OR "peripheral arterial disease"[Title/Abstract] OR "peripheral artery disease"[Title/Abstract] OR "peripheral vascular disease"[Title/Abstract]

**Covariates in SIMPLER**

Covariates included in the multivariable model were age (continuous in year), sex, body mass index (height divided by weight squared), education level (≤9, 10 to 12, >12 years), smoking (never smoker, past smoker with <20, 20-39 and ≥ 40 pack-years and current smoker with <20, 20-39 and ≥ 40 pack-years), physical activity (0-10, 11-30 and 31-60 and >60 minutes per day of walking or cycling), diet quality (categories by quartiles), and baseline history of hypertension, hypercholesterolemia, and diabetes as obtained from the 2008 health questionnaire and 2009 lifestyle questionnaire. Diet quality was assessed by a modified version of the Dietary Approaches to Stop Hypertension diet. Diet quality was assessed by a modified version of the Dietary Approaches to Stop Hypertension diet. This dietary quality score included fruits, vegetables, nuts and legumes, whole grains, and low‐fat dairy products as healthy components and red and processed meat and sweetened beverages as unhealthy components (1). Individuals were assigned a score from 1 to 5 according to the quintiles of consumption of each food and the scores were summed to create a diet score (7 to 35). A high score indicates a high adherence to the modified Dietary Approaches to Stop Hypertension diet pattern.

**Covariates in the UK Biobank study**

| **Covariate** | **Definition** |
| --- | --- |
| Age | Age of the participant on the day they attended an Initial Assessment Centre, truncated to whole year. |
| Sex | Female and male |
| Ethnicity | White and other ethnicities |
| Education | With and without college/university degree |
| Townsend deprivation index | It is estimated using the method mentioned online (<https://biobank.ndph.ox.ac.uk/showcase/label.cgi?id=76>). It has been categorized into three categories: high, moderate, and low deprivation. |
| Body mass index | Body mass divided by the square of the body height, and it is expressed in units of kg/m² |
| Smoking status | Categorical variables with 2 groups: ever and never. |
| Physical activity | Categorical variables with 3 groups: high, moderate, and low. |
|  | Over the past two weeks, how often have you had little interest or pleasure in doing things? Score: 0 (not at all); 1 (several days); 2 (more than half the days); 3 (nearly every day) |
| Diet | A dietary pattern including 7 common foods was used to assess the diet quality according to the American dietary guidelines (2). Dietary intake information was collected by a food frequency questionnaire. One diet point was given if the intakes were met for a) fruits ≥3 times/day; b) vegetables ≥3 times/day; c) fish ≥2 times/week; d) whole grains ≥3 times/day; e) refined grains ≤1.5 times/day; f) processed meats ≤1 times/week; and g) unprocessed red meats ≤2.5 times/week. The diet score ranged from 0 to 7, and a diet score ≥4 indicated a high adherence to a healthy dietary pattern. |
| Baseline hypertension | Yes or no |
| Baseline hypertension | Yes or no |
| Baseline hypercholesterolemia | Yes or no |

**Refs:**

(1) Yuan S, Bruzelius M, Håkansson N, Åkesson A, Larsson SC. Lifestyle factors and venous thromboembolism in two cohort studies. Thrombosis Research. 2021. doi: https://doi.org/10.1016/j.thromres.2021.03.024

(2) Mozaffarian D, Appel LJ, Van Horn L. Components of a cardioprotective diet: new insights. Circulation 2011; 123(24): 2870-91.

# **Supplementary Table 1. Diagnostic information for peripheral artery disease in SIMPLER**

| **ICD codes** | | |
| --- | --- | --- |
| **ICD 9** | **ICD10** | **Definition** |
| 440.0 | I70.0 | Atherosclerosis of aorta |
| 440.2 | I70.2 | Atherosclerosis of native arteries of the extremities |
| 440.3 | I70.3 | Atherosclerosis of unspecified type of bypass graft of the extremities |
| 440.4 | I70.4 | Atherosclerosis of autologous vein bypass graft of the extremities |
| NA | I70.5 | Atherosclerosis of nonautologous biological bypass graft(s) of the extremities |
| NA | I70.6 | Atherosclerosis of nonbiological bypass graft of the extremities |
| NA | I70.7 | Atherosclerosis of other type of bypass graft of the extremities |
| 440.9 | I70.9 | Other and unspecified atherosclerosis |
| 443.9 | I73.9 | Peripheral vascular disease, unspecified |
| **Procedure codes** | | |
| PDF10, PDF30, PDH10, PDH20, PDH21, PDH22, PDH23, PDH24, PDH30, PDH35, PDH99, PDN10, PDN30, PDP10, PDP30, PDQ10, PDQ30, PDW99, PEF10, PEF11, PEF12, PEH10, PEH11, PEH12, PEH20, PEH30, PEN10, PEN11, PEN12, PEP10, PEP11, PEP12, PEQ10, PEQ11, PEQ12, PEW99, PFH10, PFH20, PFH21, PFH22, PFH23, PFH24, PFH25, PFH26, PFH27, PFH28, PFH29, PFH99, PFN10, PFP10, PFQ10, PFQ30, PFW99, PGH20, PGH21, PGH22, PGH23, PGH30, PGH31, PGH40, PGH99, PGW99 | | |

# **Supplementary Table 2. Diagnostic information for peripheral artery disease in UK Biobank**

| **ICD-9 diagnosis** | **ICD-10 diagnosis** | **Self-report** |
| --- | --- | --- |
|  |  |  |
| 443.8, 443.9 | I73.8, I73.9 | 20002 |

# **Supplementary Table 3. Baseline characteristics of 70,116 participants by alcohol consumption status in SIMPLER**

|  | **Alcohol consumption** | | | |  | **Total** |
| --- | --- | --- | --- | --- | --- | --- |
|  | **Never drinker** | **Current drinker*** |  |  |  |  |
| **Characteristics** |  | **Light** | **Moderate** | **High** | **Heavy** |  |
| Individuals | 6477 | 47731 | 12244 | 3303 | 361 | 70116 |
| Age, mean±SD, years | 66.3±9.2 | 59.9±9.1 | 57.9±8.7 | 58.6±8.9 | 60.8±9.4 | 60.1±9.3 |
| Alcohol intake range gram/day | 0 | 0.1-12.0 | 12.1-24.0 | 24.1-48.0 | 48.1-310.1 | 0-310.1 |
| Alcohol in gram per day | 0 | 4.7±3.3 | 16.5±3.3 | 30.8±5.8 | 65.6±21.9 | 7.9±9.0 |
| Male, % | 32 | 46.6 | 81.6 | 91.5 | 93.4 | 53.7 |
| Body mass index, mean±SD, kg/m^2^ | 25.7±4.1 | 25.4±3.7 | 25.3±3.2 | 25.5±3.2 | 25.4±3.6 | 25.4±3.6 |
| Post-secondary education, % | 8.9 | 17.4 | 25.5 | 28.1 | 23.3 | 18.6 |
| Hypertension, % | 23.6 | 20.0 | 18.0 | 19.2 | 17.5 | 19.9 |
| Hypercholesterolemia, % | 7.5 | 9.7 | 10.9 | 11.9 | 11.1 | 9.8 |
| Diabetes, % | 6.7 | 4.1 | 3.4 | 3.3 | 5.8 | 4.2 |
| Smoking status a, % |  |  |  |  |  |  |
| Never smoker | 84 | 45.8 | 32.9 | 26.5 | 22.2 | 46 |
| Past smoker | 8.3 | 31.1 | 42.9 | 46.5 | 41 | 31.9 |
| Current smoker | 7.7 | 23.1 | 24.2 | 27 | 36.8 | 22.1 |
| Physical activity a, % |  |  |  |  |  |  |
| <10 mins/day | 10.2 | 7.1 | 7.7 | 10.2 | 11.4 | 7.6 |
| 10-30 mins/day | 14.5 | 14.7 | 15.7 | 15.8 | 13.3 | 14.9 |
| 31-60 mins/day | 32.6 | 37.5 | 36.5 | 33.9 | 31.6 | 36.6 |
| >60 mins/day | 42.7 | 40.8 | 40.1 | 40.1 | 43.8 | 40.9 |
| mDASH score, mean±SD | 17.2±3.6 | 17.5±3.6 | 17.2±3.5 | 17.0±3.5 | 16.4±3.9 | 17.4±3.6 |

mDASH, modified version of the Dietary Approaches to Stop Hypertension; SD, standard deviation; SIMPLER, Swedish Infrastructure for Medical Population-Based Life-Course and Environmental Research. *Four categories of alcohol intake: light (0.1-7 drinks/week), moderate (7.1-14 drinks/week), high (14.1-28 drinks/week), and heavy (>28 drinks/week)

# **Supplementary Table 4. Associations of alcohol consumption with incident PAD in SIMPLER**

| **Alcohol consumption** | **Cases** | **Total** | **HR** | **95% CI** | ***P*** |
| --- | --- | --- | --- | --- | --- |
| **Model 1** |  |  |  |  |  |
| Never | 272 | 6477 | Ref | - | - |
| Light (0.1-7 drinks/week) | 1708 | 47,731 | 1.05 | 0.92-1.19 | 0.486 |
| Moderate (7.1-14 drinks/week) | 433 | 12,244 | 1.06 | 0.91-1.25 | 0.461 |
| High (14.1-28 drinks/week) | 121 | 3303 | 1.08 | 0.87-1.35 | 0.473 |
| Heavy (>28 drinks/week) | 20 | 361 | 1.69 | 1.07-2.68 | 0.024 |
| **Model 2** |  |  |  |  |  |
| Never | 272 | 6477 | Ref | - | - |
| Light (0.1-7 drinks/week) | 1708 | 47,731 | 0.83 | 0.72-0.95 | 0.006 |
| Moderate (7.1-14 drinks/week) | 433 | 12,244 | 0.81 | 0.69-0.96 | 0.015 |
| High (14.1-28 drinks/week) | 121 | 3303 | 0.80 | 0.64-1.01 | 0.061 |
| Heavy (>28 drinks/week) | 20 | 361 | 1.20 | 0.75-1.9 | 0.444 |
| **Model 3** |  |  |  |  |  |
| Never | 272 | 6477 | Ref | - | - |
| Light (0.1-7 drinks/week) | 1708 | 47,731 | 0.83 | 0.74-0.93 | 0.006 |
| Moderate (7.1-14 drinks/week) | 433 | 12,244 | 0.82 | 0.71-0.94 | 0.019 |
| High (14.1-28 drinks/week) | 121 | 3303 | 0.81 | 0.67-1.01 | 0.071 |
| Heavy (>28 drinks/week) | 20 | 361 | 1.22 | 0.69-2.15 | 0.391 |

CI, confidence interval; HR, hazard ratio; PAD, peripheral artery disease; SIMPLER, Swedish Infrastructure for Medical Population-Based Life-Course and Environmental Research. Using age as the underlying time scale, model 1 was adjusted for sex; model 2 was adjusted for sex, body mass index, education level, smoking status, physical activity, and diet quality; and model 3 (the main analysis) was adjusted for sex, body mass index, education level, smoking status, physical activity, diet quality, and baseline history of hypertension, diabetes, and hypercholesterolemia.

# **Supplementary Table 5. Baseline characteristics of 405,406 participants by alcohol consumption status in the UK Biobank study**

|  | **Alcohol consumption** | | | |  | **Total** |
| --- | --- | --- | --- | --- | --- | --- |
|  | **Never drinker** | **Current drinker*** | | | |  |
| **Characteristics** |  | **Light** | **Moderate** | **High** | **Heavy** |  |
| Individuals | 33,322 | 185,351 | 104,318 | 64,738 | 17,677 | 405,406 |
| Age, mean±SD, years | 57.0±8.4 | 56.7±8.1 | 56.4±8.0 | 56.4±7.9 | 56.0±7.8 | 56.6±8.1 |
| Alcohol intake range gram/day | 0 | 0.2-12.0 | 12.1-24.0 | 24.1-48.0 | 48.1-732 | 0-732 |
| Alcohol in gram per day | 0 | 7.3±3.9 | 20.1±3.9 | 38.2±7.5 | 78.0±26.3 | 18.0±18.8 |
| Male, % | 27.8 | 34.3 | 55.1 | 74.2 | 88,1 | 47.8 |
| Body mass index, mean±SD, kg/m^2^ | 28.2±5.6 | 27.0±4.7 | 27.0±4.3 | 27.7±4.2 | 28.2±4.4 | 27.2±4.6 |
| With college/university degree, % | 24.5 | 34.8 | 36.9 | 34.0 | 27.6 | 34.1 |
| Hypertension, % | 32.5 | 25.3 | 26.3 | 31.6 | 39.0 | 27.7 |
| Diabetes, % | 10.1 | 4.4 | 3.9 | 4.6 | 5.3 | 4.8 |
| Smoking status a, % |  |  |  |  |  |  |
| Never smoker | 26.2 | 38.1 | 51.0 | 62.3 | 72.4 | 45.8 |
| Past/current smoker | 73.8 | 61.9 | 49.0 | 37.7 | 27.6 | 54.2 |
| Physical activity a, % |  |  |  |  |  |  |
| Irregular physical activity | 26.9 | 20.4 | 18.7 | 19.5 | 22.9 | 20.5 |
| Regular physical activity | 73.1 | 79.6 | 81.3 | 80.5 | 77.1 | 79.5 |
| Adherence to a healthy diet, % | 71.2 | 76.0 | 71.7 | 62.6 | 48.6 | 71.2 |

SD, standard deviation.

*Four categories of alcohol intake: light (0.1-7 drinks/week), moderate (7.1-14 drinks/week), high (14.1-28 drinks/week), and heavy (>28 drinks/week).

# **Supplementary Table 6. Associations of alcohol consumption with incident PAD in the UK Biobank study**

| **Alcohol consumption** | **Cases** | **Total** | **HR** | **95% CI** | ***P*** |
| --- | --- | --- | --- | --- | --- |
| **Model 1** |  |  |  |  |  |
| Never | 443 | 32,879 | Ref | - | - |
| Light (0.1-7 drinks/week) | 1571 | 183,780 | 0.62 | 0.55-0.69 | <0.001 |
| Moderate (7.1-14 drinks/week) | 1089 | 103,229 | 0.65 | 0.58-0.73 | <0.001 |
| High (14.1-28 drinks/week) | 957 | 63,781 | 0.83 | 0.73-0.93 | 0.002 |
| Heavy (>28 drinks/week) | 472 | 17,205 | 1.50 | 1.31-1.72 | <0.001 |
| **Model 2** |  |  |  |  |  |
| Never | 443 | 32,879 | Ref | - | - |
| Light (0.1-7 drinks/week) | 1571 | 183,780 | 0.65 | 0.59-0.73 | <0.001 |
| Moderate (7.1-14 drinks/week) | 1089 | 103,229 | 0.63 | 0.56-0.71 | <0.001 |
| High (14.1-28 drinks/week) | 957 | 63,781 | 0.70 | 0.62-0.79 | <0.001 |
| Heavy (>28 drinks/week) | 472 | 17,205 | 1.07 | 0.93-1.23 | 0.335 |
| **Model 3** |  |  |  |  |  |
| Never | 443 | 32,879 | Ref | - | - |
| Light (0.1-7 drinks/week) | 1571 | 183,780 | 0.71 | 0.64-0.79 | <0.001 |
| Moderate (7.1-14 drinks/week) | 1089 | 103,229 | 0.70 | 0.62-0.79 | <0.001 |
| High (14.1-28 drinks/week) | 957 | 63,781 | 0.78 | 0.69-0.88 | <0.001 |
| Heavy (>28 drinks/week) | 472 | 17,205 | 1.17 | 1.02-1.34 | 0.030 |

CI, confidence interval; HR, hazard ratio; PAD, peripheral artery disease. Using age as the underlying time scale, model 1 was adjusted for sex; model 2 was adjusted for sex, body mass index, education level, smoking status, physical activity, and diet quality; and model 3 (the main analysis) was adjusted for sex, body mass index, education level, smoking status, physical activity, diet quality, and baseline history of hypertension, diabetes, and hypercholesterolemia.

# **Supplementary Table 7. Association between alcohol consumption and PAD risk by baseline age, sex, and smoking status in SIMPLER**

| **Group** | **HR** | **95% CI** | ***P*** |
| --- | --- | --- | --- |
| **Baseline age <60** |  |  |  |
| Never | Ref |  |  |
| Light | 0.92 | 0.63-1.36 | 0.684 |
| Moderate | 0.77 | 0.51-1.17 | 0.221 |
| High | 0.81 | 0.5-1.31 | 0.382 |
| Heavy | 1.15 | 0.49-2.66 | 0.749 |
| **Baseline age >=60** |  |  |  |
| Never | Ref |  |  |
| Light | 0.84 | 0.72-0.97 | 0.019 |
| Moderate | 0.93 | 0.76-1.12 | 0.440 |
| High | 0.87 | 0.66-1.15 | 0.326 |
| Heavy | 1.28 | 0.73-2.26 | 0.390 |
| **Men** |  |  |  |
| Never | Ref |  |  |
| Light | 1.30 | 0.99-1.72 | 0.060 |
| Moderate | 1.29 | 0.96-1.72 | 0.088 |
| High | 1.23 | 0.88-1.71 | 0.227 |
| Heavy | 2.03 | 1.21-3.42 | 0.007 |
| **Women** |  |  |  |
| Never | Ref |  |  |
| Light | 0.69 | 0.58-0.81 | 0.000 |
| Moderate | 0.58 | 0.43-0.79 | 0.001 |
| High | 0.77 | 0.42-1.43 | 0.411 |
| Heavy | - | - | - |
| **Never smoker** |  |  |  |
| Never | Ref |  |  |
| Light | 0.90 | 0.76-1.06 | 0.222 |
| Moderate | 0.96 | 0.73-1.25 | 0.762 |
| High | 1.09 | 0.70-1.72 | 0.696 |
| Heavy | 0.95 | 0.24-3.86 | 0.947 |
| **Ever smoker** |  |  |  |
| Never | Ref |  |  |
| Light | 0.73 | 0.57-0.94 | 0.014 |
| Moderate | 0.71 | 0.54-0.93 | 0.013 |
| High | 0.67 | 0.48-0.92 | 0.014 |
| Heavy | 1.10 | 0.65-1.86 | 0.727 |

CI, confidence interval; HR, hazard ratio; PAD, peripheral artery disease. Using age as the underlying time scale, the analysis was adjusted for age, sex, body mass index, education level, smoking, physical activity, diet quality, and baseline hypertension, diabetes, and hypercholesterolemia.

# **Supplementary Table 8. Association between alcohol consumption and PAD risk by baseline age, sex, and smoking status in the UK Biobank**

| **Group** | **HR** | ***P*** |
| --- | --- | --- |
| **Baseline age <60** |  |  |
| Never | Ref |  |
| Light | 0.66 (0.53, 0.83) | <0.001 |
| Moderate | 0.60 (0.47, 0.77) | <0.001 |
| High | 0.71 (0.56, 0.91) | 0.007 |
| Heavy | 1.12 (0.86, 1.47) | 0.396 |
| **Baseline age >=60** |  |  |
| Never | Ref |  |
| Light | 0.65 (0.58, 0.74) | <0.001 |
| Moderate | 0.64 (0.56, 0.73) | <0.001 |
| High | 0.70 (0.61, 0.80) | <0.001 |
| Heavy | 1.04 (0.88, 1.22) | 0.669 |
| **Men** |  |  |
| Never | Ref |  |
| Light | 0.73 (0.62, 0.86) | <0.001 |
| Moderate | 0.69 (0.58, 0.81) | <0.001 |
| High | 0.74 (0.63, 0.88) | 0.001 |
| Heavy | 1.17 (0.98, 1.40) | 0.087 |
| **Women** |  |  |
| Never | Ref |  |
| Light | 0.59 (0.51, 0.68) | <0.001 |
| Moderate | 0.58 (0.48, 0.69) | <0.001 |
| High | 0.78 (0.63, 0.97) | 0.023 |
| Heavy | 0.91 (0.58, 1.45) | 0.698 |
| **Never smoker** |  |  |
| Never | Ref |  |
| Light | 0.62 (0.54, 0.72) | <0.001 |
| Moderate | 0.60 (0.52, 0.70) | <0.001 |
| High | 0.69 (0.59, 0.80) | <0.001 |
| Heavy | 1.04 (0.88, 1.23) | 0.622 |
| **Ever smoker** |  |  |
| Never | Ref |  |
| Light | 0.72 (0.61, 0.86) | <0.001 |
| Moderate | 0.69 (0.57, 0.85) | <0.001 |
| High | 0.70 (0.56, 0.88) | 0.002 |
| Heavy | 0.98 (0.70, 1.36) | 0.896 |

CI, confidence interval; HR, hazard ratio; PAD, peripheral artery disease. Using age as the underlying time scale, the analysis was adjusted for sex, ethnicity, body mass index,, education level, smoking status, physical activity, adherence to a healthy diet, baseline hypertension, diabetes, and hypercholesterolemia.

# **Supplementary Table 9. Quality assessment of included 9 studies.**

| **Studies** | **Selection** | | | | **Comparability** | **Outcome** | | | **Total (9/9)** |
| --- | --- | --- | --- | --- | --- | --- | --- | --- | --- |
|  | **Representativeness of the exposed cohort** | **Selection of the nonexposed cohort** | **Ascertainment of exposure** | **Outcome of interest was not present at start of study** | **Comparability of cohorts on the basis of the design or analysis** | **Assessment of outcome** | **Efficient follow-up time** | **Adequacy of follow up** |  |
| Camargo et al. 1997 | 1 | 1 | 1 | 1 | 2 | 0 | 1 | 1 | 8 |
| Vliegenthart R etal., 2002 | 0 | 1 | 1 | 0 | 1 | 0 | 0 | 0 | 3 |
| Ciccarone E et al., 2003 | 0 | 1 | 1 | 0 | 1 | 1 | 0 | 0 | 4 |
| Xie X et al., 2010 | 0 | 1 | 0 | 0 | 2 | 1 | 0 | 0 | 4 |
| Desormais I et al., 2015 | 0 | 1 | 1 | 0 | 2 | 1 | 0 | 0 | 5 |
| Ogilvie RP et al., 2017 | 1 | 1 | 0 | 1 | 2 | 1 | 1 | 0 | 7 |
| Bell S et al., 2017 | 1 | 1 | 1 | 1 | 1 | 1 | 1 | 1 | 8 |
| López-Laguna N et al., 2018 | 0 | 1 | 1 | 1 | 2 | 1 | 1 | 0 | 7 |
| Chen GC et al., 2021 | 1 | 1 | 0 | 1 | 2 | 1 | 1 | 0 | 7 |

**Supplementary Figure 1. The U-shaped association between alcohol consumption and PAD risk in SIMPLER**


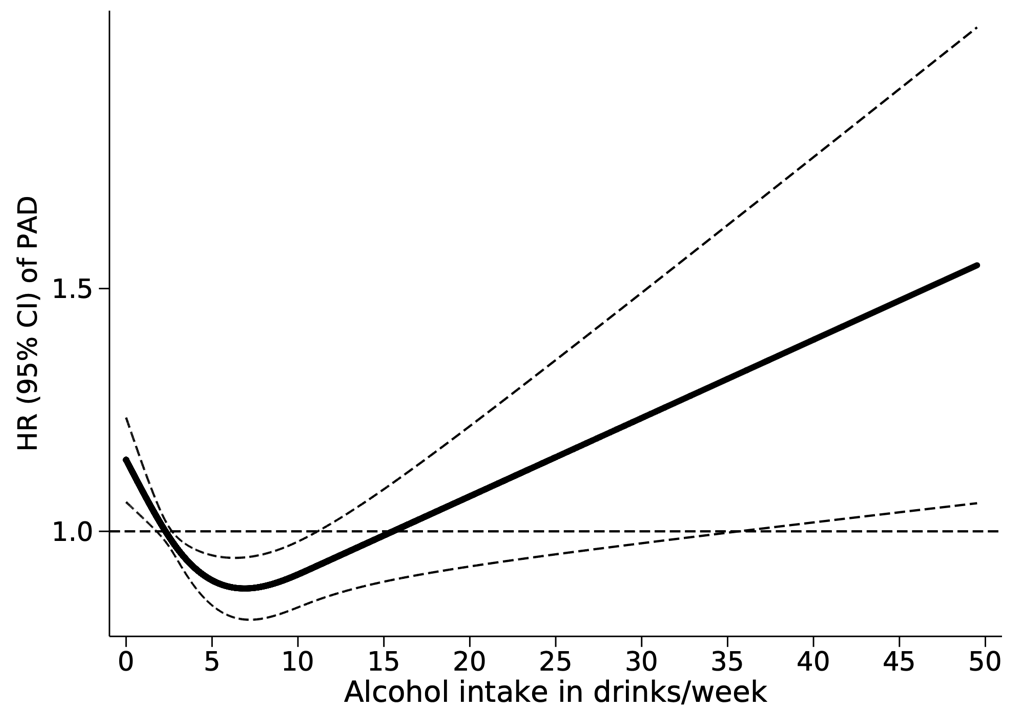


CI, confidence interval; HR, hazard ratio; PAD, peripheral artery disease; SIMPLER, Swedish Infrastructure for Medical Population-Based Life-Course and Environmental Research. Using age as the underlying time scale, the associations were adjusted for sex, body mass index, education level, smoking, physical activity, diet quality, and energy intake. One drink corresponds to 12 g ethanol.

# **Supplementary Figure 2. The U-shaped association between alcohol consumption and PAD risk in the UK Biobank study**


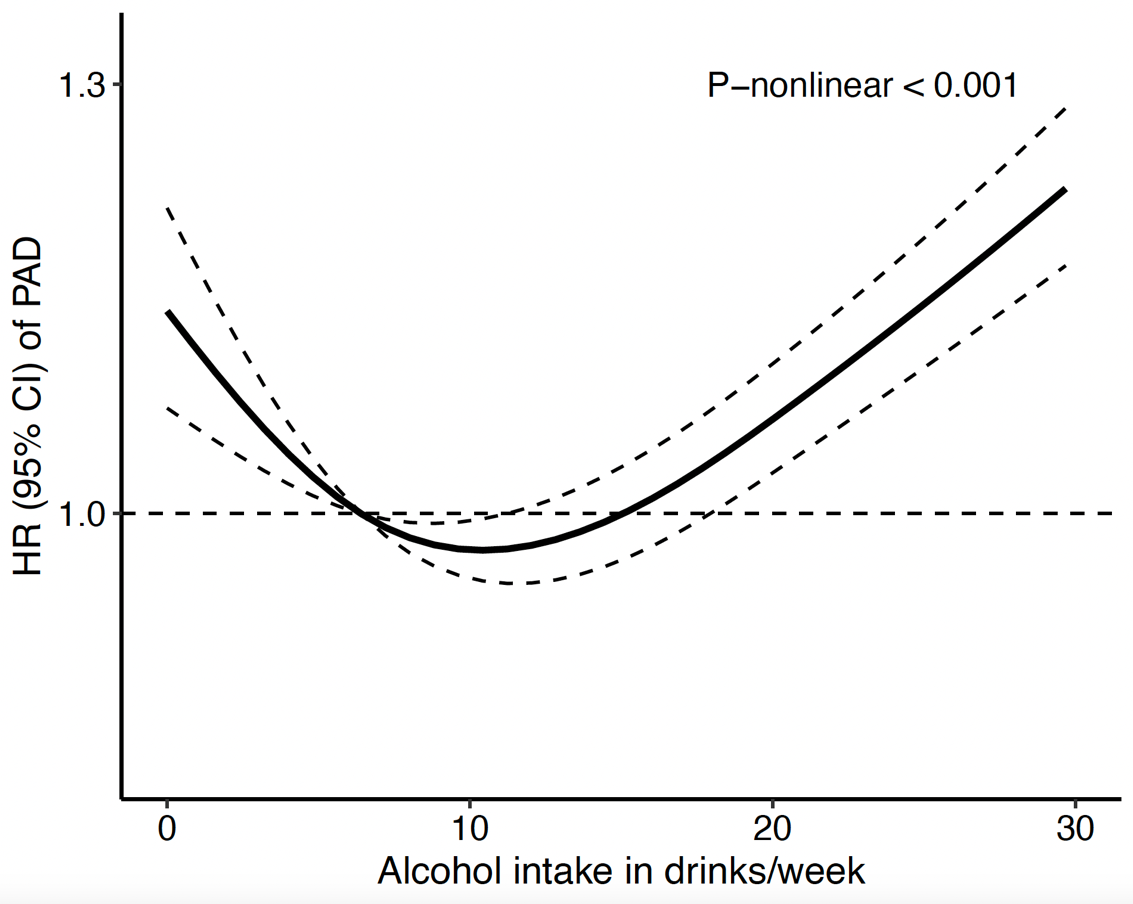


CI, confidence interval; HR, hazard ratio; PAD, peripheral artery disease. Using age as the underlying time scale, the association was adjusted for sex, body mass index, education level, smoking status, physical activity, diet quality, and baseline history of hypertension, diabetes, and dyslipidemia.

# **Supplementary Figure 3. Flow chart of systematic review**


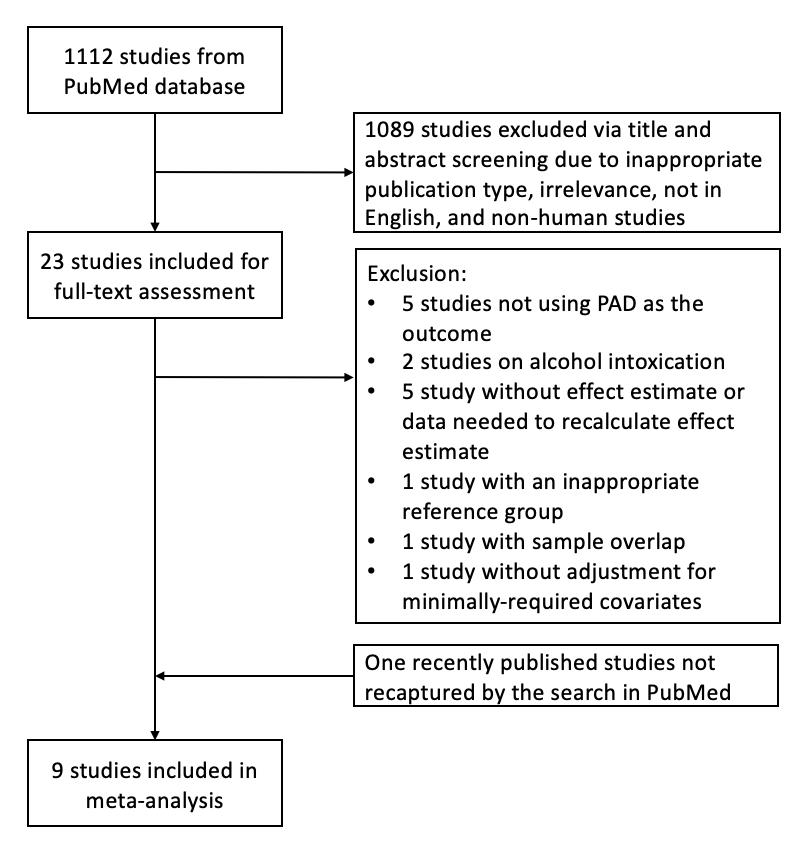


PAD, peripheral artery disease.

**Supplementary Figure 4. Funnel plot and results of Egger's and Begg's tests**


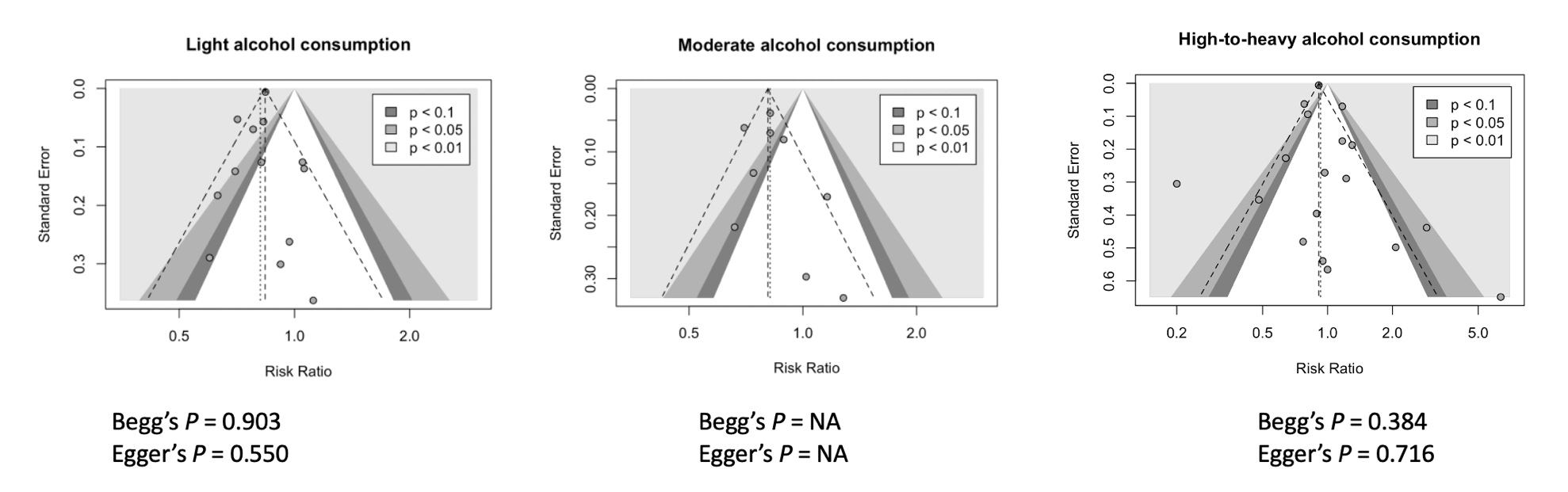

Supplement: zwae142_Supplementary_Data [file zwae142_supplementary_data.docx]
